# A scoping review of arts in mental health policy in the United States

**DOI:** 10.3389/fpubh.2025.1562990

**Published:** 2025-05-05

**Authors:** Alexandra K. Rodriguez, Jennifer L. Kuo, Cris Sanhueza, Gray Davidson Carroll, Courtney Pyche, Jane Morgan-Daniel, George Hack, Jill Sonke

**Affiliations:** ^1^University of Florida Center for Arts in Medicine, Gainesville, FL, United States; ^2^University of Florida College of Public Health and Health Professions, Gainesville, FL, United States; ^3^University of Florida Health Science Center Libraries, Gainesville, FL, United States

**Keywords:** arts in mental health, mental health, arts in health, arts and health, arts and health policy, mental health policy

## Abstract

The mental health crisis in the United States has been exacerbated with the emergence of the loneliness epidemic and resurgence of mental health inequities. To address the scope of this crisis comprehensively and equitably, a socioecological, cross-sectoral approach is necessary. While arts in mental health strategies have been employed internationally and nationally for preventative and rehabilitative mental health support, there remains limited knowledge of policy in the US to sustain and expand arts in mental health practices. Subsequently, this review sought to understand what priorities and strategies are employed in public health policies that seek to engage the arts to address mental health in the United States. Fourteen databases — inclusive of Embase, PsycINFO, PubMed, Scopus, and PolicyFile — were searched alongside a comprehensive grey literature search. Included documents were originated by a US organization or agency, included a mode and form of arts participation, had a focus on mental health, maintained a public health purview, pertain to the United States, and can be considered a policy document. Of 4,958 identified documents, 29 met inclusion criteria and were included. Following extraction, the evidence revealed several salient results: (a) the relative nascency of arts in mental health policy documents in the United States; (b) that policy recommendations primarily center on creating sustained, collective action and leveraging funding; and (c) that the arts sector alongside the arts and health sector are primarily leading policy work. Current momentum in the United States offers a “policy window” as there is alignment, as evidenced in this review, amongst national policy makers, the prevailing mental health crisis, and opportunities for arts in mental health policies as a viable solution. As such, this work can be mobilized to strategize how to best engage or promote the engagement of local artists, mental health practitioners, arts in mental health researchers, and policy makers in the development of arts in mental health policies moving forward. Future work should seek to intentionally build on areas of sustained effort to effectively catalyze future work towards developing legislative, regulative, or even litigative cross-sectoral, arts in mental health policies.

## Introduction

According to the National Institute of Mental Health (NIMH), greater than one in five adults in the United States (US) — about 57.8 million people — currently experience mental illness (1). Further, not only has this prevalence increased since the COVID-19 pandemic, but it has also disproportionately impacted minoritized communities (2). As evidenced by the US Surgeon General’s recent advisory, the expansive scope of this issue can be linked to an epidemic of loneliness and social isolation (3). This national advisory highlights the limitations of combatting this crisis solely through a biomedical model. To address the scope of mental illness comprehensively and equitably within the US, while further bolstering mental health, a socioecological, cross-sectoral approach is necessary.

Arts participation, as defined by Sonke et al. (4), considers culturally inclusive modes and forms of engagement which can be leveraged across all levels of the social ecological model to support mental health (5). As evidenced by the World Health Organization (WHO), participation in the arts offers opportunities to support mental health through a strength-based approach while also acting as a rehabilitative modality for mental illness (6). The WHO also advocates for this intersectional approach by noting that “stronger pathways between the arts, health and social care can provide creative solutions to help to achieve the Health 2020 targets and the Sustainable Development Goals” [(7), p.1]. The evidence at the intersection of the arts and mental health can be considered across a continuum spanning arts engagement in communities, public health practice, and clinical settings. Notably, epidemiological studies have considered cohort data from both the US and United Kingdom and have found that arts engagement was associated with a greater ability to cope with mental health issues in everyday life (8). More specifically, data from 12,055 adults in the US Health and Retirement Study found that group arts participation has been associated with multiple aspects of wellbeing including positive affect, life satisfaction, perceived mastery, and purpose in life (8, 9). Further, evidence has shown that adults who are over the age of 50 who visited cultural venues every few months had a 32% lower risk of developing depression over ten years (8, 10). Further, the arts have even been shown to support mental health in times of crises as evidenced during the COVID-19 pandemic (11). Despite the demonstrated efficacy of the arts in supporting mental health, there remains limited infrastructure in the US to sustain and expand arts in mental health practices.

Efforts in the US to create pathways at scale for cross-sector collaboration between artists and mental health professionals are nascent yet promising. Notably, the National Endowment for the Arts (NEA) and the US Department of Health and Human Services (HHS) have established an Interagency Working Group on Arts, Health, and Civic Infrastructure which seeks to “foster exchanges of insights and information about arts and cultural resources and strategies across federal agencies, with the goal of helping to improve the health and well-being of individuals and communities” (12). Other cross-agency efforts have also acted to promote the advancement and sustainability of this work. Some recent examples include the Federal Interagency Task Force on the Arts and Human Development (13), Sound Health: An NIH-Kennedy Center Partnership (14), and NEA partnerships with the Centers for Disease Control and Prevention (CDC) and the CDC Foundation (15, 16). The NEA also co-hosted a summit alongside the White House Domestic Policy Council — Healing, Bridging, Thriving: A Summit on Arts and Culture in our Communities — which brought together national arts leadership and health leadership on a nationally broadcast stage to discuss how the arts can be leveraged to address our country’s multi-dimensional needs (17). While these notable efforts have progressed arts in mental health work nationally, policy achievements remain limited.

Policy — whether legislation, regulation, litigation, or other forms inclusive of policies which may be organizational or do not have the force of law (18) — offers a mechanism by which to create arts-based mental health solutions at scale. Internationally, countries have already been mobilizing arts in public health policies to employ this practice at scale (19). Notably, a review by Dow et al. (19) which considered arts in public health policies internationally found that the most promising current practices, such as forms of social prescribing, are those developed through collaboration by both health and arts sectors. In the US, policy momentum has been supported by President Biden’s Executive Order on Promoting the Arts, the Humanities, and Museum and Library Services (20, 21). Within his executive order, President Biden asserted that…

*Under my Administration, the arts, the humanities, and museum and library services will be integrated into strategies, policies, and programs that advance the economic development, well-being, and resilience of all communities, especially those that have historically been underserved. [They] will be promoted and expanded to strengthen public, physical, and mental health; wellness; and healing, including within military and veteran communities* (20, 21).

This degree of support has presented a “policy window” for policy development at this intersection as there is alignment amongst a prevalent need, political momentum, and a viable solution (22). However, it is important to note that despite bipartisan support for arts and health across past administrations, the most recent administration openly threatens this bipartisan commitment to arts, culture, and wellbeing — weakening a once widely open policy window (23, 24).

Despite a high-level review of arts in public health policy internationally (19), there is no current literature delving into such policy within the US, especially as it relates to priorities and strategies employed by US arts in mental health policy. This is of particular importance given the role that policy at this intersection can play in supporting health equity (5). Subsequently, the objective of this scoping review was to assess the extent of the literature which considers current arts and public health policies seeking to address mental health in the US. Given the current gap in the literature, our team sought to utilize a scoping review to address the following research question: *What priorities and strategies are employed in public health policies that seek to engage the arts to address mental health in the United States?*

## Methods

To effectively investigate policy at this intersection, a scoping review design was chosen given its ability to comprehensively synthesize evidence from a broader scope than systematic reviews allow (25). Further, this approach is ideal for emerging fields and disciplines, such as arts in mental health, as it allows for an array of study designs and sources to be included which furthers the extent to which the review process can identify gaps in the literature and areas which warrant further investigation (26). A preliminary search of Campbell Collaboration Library, PROSPERO, JBI Evidence Synthesis, Cochrane Database of Systematic Reviews, BioMed Central Reviews, Cochrane Public Health Review Group, and Open Science Framework (OSF) was conducted, and no current or underway systematic reviews or scoping reviews on the topic were identified. This review was conducted in accordance with the Joanna Briggs Institute’s (JBI) Manual for Evidence Synthesis for scoping reviews to ensure a rigorous and systematic process for literature searching, screening, and extracting (26). Additionally, a protocol for the review was registered with Open Science Framework and underwent one revision iteration to further refine the extraction criteria and add additional authors (27). The reporting structure of the review was guided by the PRISMA Extension for Scoping Reviews (PRISMA-ScR) Checklist (28). Moreover, the definitions utilized for each construct considered in this review are detailed in Table 1.

**Table 1:** Working definitions.

Construct	Definition
Arts participation	Modes: “Attending live arts and cultural events and activities; Creating, practicing, performing, and sharing art; Participating in social, civic, spiritual, and cultural arts practices; Consuming arts via electronic, digital, or print media; Learning in, through, and about the arts” Forms: “Dance/Movement (such as aerial, ballet, ballroom, ceremonial, contemporary, cultural, hip-hop, jazz, step, or tap) Literary Arts (such as storytelling, fiction, nonfiction, short stories, memoir, screenwriting, poetry, literature for children, or graphic novels) Media (such as film, animation, video, or other work at the intersection of technology, aesthetics, storytelling, and digital cultures) Music (such as rap, choral, contemporary, experimental, gospel, instrumental, hip-hop, classical, chanting, rock, electronic, drumming, pop, world, or jazz) Theater/Performance (such as theater, musical theater, devised theater, puppetry, performance art, ritual, opera, spoken word, stage design, circus arts, comedy) Visual Arts, Craft, and Design (such as illustration, painting, drawing, collage, printmaking, installation, photography, gardening, sculpture, video art, street art, pottery, glass, jewelry, metalworking, textiles, fashion, culinary arts, and graphic, floral, architectural, environmental, or industrial design)” [(4), p.9]
Policy	Policy making bodies Governmental Federal State Local Nongovernmental Private Institutional Types of policies Legislation Regulation Litigation Other: Presidential and gubernatorial executive orders are legally binding and allow for rapid policy change Some policies do not have the force of law (e.g., guidance documents produced by federal, state, or local agencies) May develop policies to be applied by institutions [(18), p.10S]
Mental health	“Mental health includes our emotional, psychological, and social well-being. It affects how we think, feel, and act. It also helps determine how we handle stress, relate to others, and make healthy choices. Mental health is important at every stage of life, from childhood and adolescence through adulthood.” (72)

### Inclusion and exclusion criteria

The Participants, Context, Concept framework was employed to specify the review’s inclusion and exclusion criteria as this is the recommended framing for scoping reviews per JBI guidelines (26). See Table 2 for further details. Regarding source types, this review considered any policy-based documents such as legislative documents, policy briefs, advisory briefs, policy-based white papers, health plans, government reports, and policy frameworks.

**Table 2:** Applied participants, context, concept framework.

Inclusion	Exclusion
Participants	
Any US organization or agency that is engaged either individually or as part of a collective in policy related activity	No US organization or agency that is engaged either individually or as part of a collective in policy related activity
Concept	
Includes a mode or form of art as defined by Sonke et al. (4) Focuses on mental health, as defined by the CDC (72) Public Health focus rather than a clinical focus	Does not include a mode or form of art as defined by Sonke et al. (4) Does not focus on mental health, as defined by the CDC (72) Does not have a public health focus rather than a clinical focus
Context	
A policy document, as defined by Pollack Porter et al. (18) In the US, defined as the 50 states, District of Columbia, US territories, and US commonwealths	Not in US defined as the 50 states, District of Columbia, US territories, and US commonwealths

### Data sources and search

The search strategy aimed to locate both published and unpublished articles. The initial form of the search string for art was derived from Pesata et al. (29) while that of mental health was derived from Rodriguez et al. (30). An initial limited search of PubMed, Web of Science, Policy File, Policy Commons, and Google was undertaken on March 14th, 2024, to identify articles on the topic. The text words contained in the titles and abstracts of relevant articles and the index terms used to describe the articles were used to develop a full search strategy. Additionally, the search strategy, including all identified keywords and index terms, was adapted for each included database and/or information source. Further, the reference list of each included source of evidence was screened for additional studies. Studies published in English were included as that is the predominate language in the US and is the only language capacity of the research team. There was no date parameter set as there is limited literature on this topic and the study sought to capture the full scope of work at this intersection.

The databases searched included Art and Architecture Source, CINAHL, Performing Arts Periodicals Database, PsycINFO, EMBASE, Scopus, PubMed (NCBI), Web of Science (Clarivate Analytics), PolicyFile, HeinOnline, Policy Commons: North American City Reports; Policy Commons: Global Think Tank, PAIS Index, Grants Index, and Dimensions. Sources of unpublished studies and grey literature searched included the Alliance for Health Policy, Brookings Institute Center for Health Policy, Georgetown University Health Policy Institute, National Academy of State Health Policy, UCLA Health Policy Research, National Conference of State Legislatures, Florida Legislature’s Office of Program Policy Analysis and Government Accountability, Kaiser Family Foundation, National Institute of Mental Health, National Association of State and Territorial Health Officials, National Association of City and County Health Organizations, National Alliance on Mental Illness, The Mental Health Innovation Network, WHO MiNDbank: Strategies and Plans, the Center for Arts in Medicine’s Repository for Arts and Health Resources, Google, and the Arts in Public Health Policy International Map. An example search strategy for PubMed is provided in Appendix A. In line with the practice undertaken by Dow et al. (19), a survey was used to capture any additional grey literature and further triangulate documents collected within the standard search. The survey utilized was disseminated via the University of Florida (UF)‘s Center for Arts in Medicine’s Instagram and Facebook accounts. It posed the research question, asked participants if they had any documents to recommend, and then allowed either upload or the entry of a direct link to relevant documents. This survey was deemed exempt by the UF Institutional Review Board (Protocol #: ET00022961).

### Evidence selection

Evidence selection was conducted by four experienced research associates from an arts and health research lab. Following the search, citations were uploaded into Covidence, and duplicates were removed. Then, after a pilot test with all four reviewers, each title and abstract was screened by two reviewers for assessment relative to the review’s inclusion and exclusion criteria. When disagreements occurred, while infrequent, the two reviewers met to discuss whether the article met inclusion criteria, and if a conclusion was not met, the principal investigator from the team was enlisted. Next, full texts of selected citations were each assessed by two independent reviewers. Reasons for exclusion at the full text stage were documented. Disagreements between reviewers at this final stage were resolved in the same manner as the stage prior.

### Data extraction and synthesis

Data from the included studies were extracted by two or more independent reviewers. The data extraction form which guided this process was informed by key recommended questions for policy analysis by the Centers for Disease Control and Prevention (CDC) (31). A draft extraction form is provided in Appendix B. The draft data extraction tool was revised as deemed necessary over the course of the extraction process. Modifications included eliminating extraction items that yielded the same information from sources, and further refining language for several extraction items to increase clarity — further details on these modifications can be found within the published Open Science Framework protocol revision: OSF.IO/GCH62 (27).

## Results

**Figure 1:**
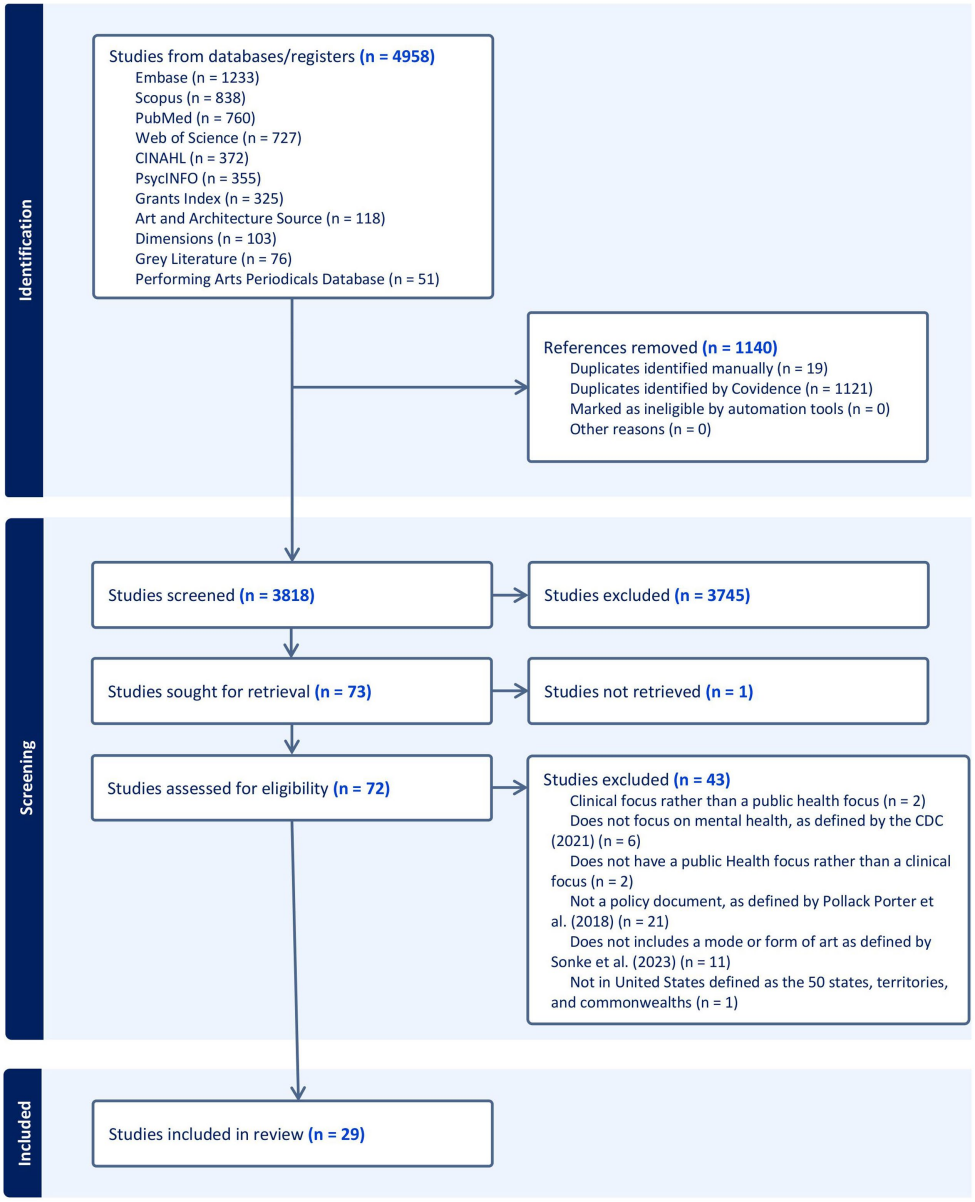
PRISMA diagram. The PRISMA diagram template was derived from Page et al. (71).

In total, 4,958 references were imported into Covidence for screening. After 1,140 duplicates were removed, 3,818 studies progressed to title and abstract screening, where 3,745 were excluded — see Figure 1. Additionally, 73 full-text articles were then assessed for eligibility, with 29 documents progressing to the data extraction stage. Additionally, the public survey garnered 13 responses, and all non-duplicates were included as a part of the grey literature count within the PRISMA diagram. To consider the breadth and depth of the extracted data, the results were considered across five primary areas: document types and organizational domains, document framing, strategies employed, field evidence, as well as funding and sustainability. Further, document framing was further subdivided to consider document scope and foci, intended audiences, and whether an equity lens was present.

### Document types and organizational domains

Sector representation in authorship and document classification were considered across all included documents. The primary authorship for each document was analyzed to consider the primary sectors represented in authorship. Notably, across all document types, there was variation in representation by sector. The arts sector was represented in primary authorship across about 59% (*n* = 23) of included documents. The arts and health sector, including organizations centered on work discretely at the intersection of arts and health, was represented across about 52% (*n* = 15) of the documents’ leading authors, while the health sector was represented across about 28% (*n* = 8). For a more specific breakdown of document type and primary authors organizational domains, see Table 3. Additionally, organizations most represented included the University of Florida Center for Arts in Medicine (UFCAM) (*n* = 5); Americans for the Arts (AFTA) (*n* = 4), ArtPlace America (*n* = 4), National Assembly of State Arts Agencies (NASAA) (*n* = 4), National Endowment for the Arts (NEA) (*n* = 4), John F. Kennedy Center for the Performing Arts (*n* = 3), National Center for Creative Aging (*n* = 3), NIH (*n* = 3), National Organization for Arts in Health (*n* = 3), US Department of Health & Human Services (*n* = 2). This is further expanded upon in Appendix C. Additionally, about 24% (*n* = 7) could be considered governmental (20, 21, 32–37), and 76% (*n* = 22) could be considered non-governmental. However, several documents were not governmental in publication but did have governmental agencies as primary partners (38–41).

**Table 3:** Documents by type, equity lens, and organizational domain of authors.

Article	Document type	Organizational domains of authors	Equity lens (Y/N)
American Art Therapy Association (42)	Policy Recommendations	Arts, Health, Military, Arts & Health	Y
Americans for the Arts (43)	Issue Brief	Arts	N
Atkins and Jacobson Blumenfeld (57)	White Paper	Arts, Health, Arts & Health	N
Biden (20, 21)	Executive Order	Municipal Leadership	Y
Bivens (52)	A Working Guide	Arts	N
Boyer (51)	Toolkit	Arts, Arts & Health	N
Cheever et al. (38)	Report	Arts, Health, Arts & Health	N
Edmonds et al. (39)	Report	Arts & Health	Y
Edwards et al. (40)	Toolkit	Arts, Health	N
Hanna et al. (32)	White Paper	Art, Health, Arts & Health	N
Hanna et al. (41)	Article	Arts, Arts & Health	N
Harlow (53)	Public Policy Published Column	Arts	Y
Iyengar et al. (33)	Strategic Framework & Agenda	Arts, Military	N
National Assembly of State Arts Agencies (45)	Policy Brief	Arts	Y
National Assembly of State Arts Agencies (44)	Strategy Sampler	Arts	N
National Assembly of State Arts Agencies (46)	Policy Brief	Arts	Y
National Institute of Health (34)	Research Plan	Health, Arts	N
National Organization of Arts and Health (59)	White Paper	Arts & Health	N
National Organization of Arts and Health (55)	Report	Arts & Health	Y
National Organization of Arts and Health (54)	Report	Arts & Health	Y
Office of Disease Prevention and Health Promotion (35)	Federal Plan	Arts, Agriculture, Commerce, Education, Environment, Health, Housing & Urban Development, Transportation	Y
Pesata et al. (47)	Advisory Brief	Academia, Arts & Health	Y
Rhode Island Department of Health (RIDOH) and The Rhode Island State Council on the Arts (RISCA) (36)	State Plan	Arts, Health	Y
Rollins (58)	Report & Blueprint for Action	Arts	N
Solis (37)	Declaration	Arts, Municipal Leadership	Y
Sonke et al. (61)	White Paper	Arts, Academia, Health, Policy, Arts & Health	Y
University of Florida Center for Arts in Medicine (48)	Advisory Brief	Arts, Health, Arts & Health	Y
University of Florida Center for Arts in Medicine (49)	Advisory Brief	Arts, Economic Development, Arts & Health	Y
University of Florida Center for Arts in Medicine (50)	Advisory Brief	Arts, Arts & Health	Y

Across the 29 documents, four document types were found: briefs, reports, toolkits/guides, and plans/agendas. Noticeably, across these categories, all included documents can be considered in the “other” category of either governmental or non-governmental policy documents as established by Pollack Porter et al. (18). Further, the most common types of documents were briefs and reports, and the most identified form of briefs (*n* = 9) were advisory or policy briefs (42–50). Toolkits, guides, plans, and agendas were less represented. The category of toolkits and guides included two toolkits (40, 51) and one field guide (52). The documents that did not adhere to a category were an executive order (20, 21), a declaration (37), public policy published column (53), and a policy journal article (41). For more detail regarding sectoral representation by document category, see Appendix D.

### Document framing

#### Document scope and foci

Both the scope and foci of the included documents informed their directed intentions. All documents addressed the role of arts in mental health, but varied in whether this was a primary or secondary focus. Most documents had a national scope (*n* = 26), while only a few considered a local or state frame (36, 37, 39). Additionally, while all included documents had an arts in mental health focus, they varied in the prioritization of this focus within the document. To contextualize this, a primary focus was determined if the concept was present in the title or aims of the document while a secondary focus was determined if the concept was present only in the narrative. The majority of documents had a primary focus on arts and health but a secondary focus on mental health which was often characterized by case building and evidence application. Of the documents that do not fall under that classification, one had a primary focus on both arts and mental health (53), several primarily focused on the arts and brain disorders (34, 38, 40), one primarily focused on mental health with a secondary focus on arts and health (42), and two had considered both the arts and mental health in a secondary manner (35, 44).

The documents also ranged in their mental health foci, as noted in Table 4. Notably, all the included articles addressed generalized mental health (*n* = 29), while the majority also discussed depression (*n* = 17) and/or anxiety (*n* = 13). Less frequently centered or mentioned were mental health topics such as Social Isolation/Loneliness (*n* = 5), Post Traumatic Stress Disorder (*n* = 4), Traumatic Brain Injuries (*n* = 4), and mental health implications of specific brain diseases such as Alzheimer’s disease and dementia (*n* = 4). In several studies, the mental health focus did not clearly align with a category. For instance, as it relates to arts engagement for mental health, several focused situated generalized mental health in the context of trauma (52), grief (47), or burnout (54).

**Table 4:** Documents by mental health foci.

Article	Mental health topic (s)
American Art Therapy Association (42)	General Mental Health, Depression
Americans for the Arts (43)	General Mental Health
Atkins and Jacobson Blumenfeld (57)	General Mental Health, Depression, Anxiety, Post Traumatic Stress Disorder, Traumatic Brain Injuries
Biden (20, 21)	General Mental Health
Bivens (52)	General Mental Health, Depression, Anxiety, Post Traumatic Stress Disorder, Traumatic Brain Injuries, Other
Boyer (51)	General Mental Health, Depression, Anxiety, Social Isolation/Loneliness
Cheever et al. (38)	General Mental Health, Depression, Anxiety, Other
Edmonds et al. (39)	General Mental Health, Depression, Anxiety, Other
Edwards et al. (40)	General Mental Health, Depression, Mental Health Implications of Brain Disease, Other
Hanna et al. (32)	General Mental Health, Depression, Mental Health Implications of Brain Disease
Hanna et al. (41)	General Mental Health, Mental Health Implications of Brain Disease
Harlow (53)	General Mental Health, Depression, Anxiety, Social Isolation/Loneliness, Other
Iyengar et al. (33)	General Mental Health, Depression, Anxiety, Post Traumatic Stress Disorder, Traumatic Brain Injuries
National Assembly of State Arts Agencies (45)	General Mental Health
National Assembly of State Arts Agencies (44)	General Mental Health, Depression, Anxiety, Post Traumatic Stress Disorder, Traumatic Brain Injuries, Other
National Assembly of State Arts Agencies (46)	General Mental Health, Depression
National Institute of Health (34)	General Mental Health
National Organization of Arts and Health (59)	General Mental Health, Depression, Anxiety, Other
National Organization of Arts and Health (55)	General Mental Health
National Organization of Arts and Health (54)	General Mental Health, Depression, Anxiety, Other
Office of Disease Prevention and Health Promotion (35)	General Mental Health, Depression, Anxiety, Other
Pesata et al. (47)	General Mental Health, Anxiety, Other
Rhode Island Department of Health (RIDOH) and The Rhode Island State Council on the Arts (RISCA) (36)	General Mental Health
Rollins (58)	General Mental Health, Depression
Solis (37)	General Mental Health
Sonke et al. (61)	General Mental Health, Depression, Anxiety, Other
University of Florida Center for Arts in Medicine (48)	General Mental Health, Social Isolation/Loneliness
University of Florida Center for Arts in Medicine (49)	General Mental Health, Social Isolation/Loneliness
University of Florida Center for Arts in Medicine (50)	General Mental Health, Social Isolation/Loneliness

Art forms and modes were considered across all included documents (4). Most documents either explicitly referenced or implied the inclusion of all art forms. However, there were four documents specific only to the art form of music (34, 38, 40). None of the documents were explicit as it pertained to modes of arts participation.

#### Intended audiences

Most of the documents clearly articulated their intended audience or audiences. The arts were represented across about 55% (*n* = 16), policy makers/government across about 52% (*n* = 15), healthcare/clinical researchers across about 45% (*n* = 13), public health across about 24% (*n* = 7), general public across about 24% (*n* = 7), education across about 24% (*n* = 7), funders/investors across about 17% (*n* = 5), and military across about 7% (*n* = 2) of the included documents. Of note was the way several of these intended audiences were contextualized. For instance, the arts sector audience was inclusive of not only artists, but also state arts agencies (44), music therapy and music medicine professionals (40), as well as arts organizations and institutions (36, 45–47, 51). Further, healthcare and clinical researchers ranged from institutions to specific types of personnel such as mental health providers (53) and neuroscientists (38, 40). Additionally, several audience types which did not conform to the above categories included civic leaders (35, 45) and arts and health researchers (32, 36, 55). For a more detailed breakdown by audience type, please see Appendix E.

#### Equity lens

To understand whether an equity lens was employed within included documents, the study considered the WHO’s definition of equity which considers it to be the “absence of unfair, avoidable or remediable differences among groups of people, whether those groups are defined socially, economically, demographically, or geographically or by other dimensions of inequality” (56). Amongst the documents that met inclusion criteria, about 55% (*n* = 16) either directly mentioned equity as a central component of the document or had a primary focus on equitable practice as evidenced by language throughout the document. Some documents, such as National Assembly of State Arts Agencies (NASAA) (45), even discussed the ability of arts engagement itself to both bridge differences in community while fostering social equity. See Table 3 for a breakdown by document.

### Strategies employed

The documents included employed aims, intended outcomes, and strategies which resulted in action plans and discrete recommendations. There was a range in the aims and intended outcomes of the documents, which aligned with the variations in scope, sector, and art form. Namely, most of the documents’ aims were directly related to the success or realization of their proposed action plans or recommendations. Additionally, action plans arose from documents characterized as either plans or agendas. Of the plans and agendas previously mentioned, three documents had a primary arts and health focus (33, 34, 36) and as such, laid forth cross-sectoral, actionable items. For example, the Rhode Island State Arts and Health Plan laid out recommendations for policy, research, and practice accompanied by a three-phase plan to mobilize arts and health research evidence with a vision toward “fully integrated and sustainable arts and health systems that build on the State’s rich creative capital and innovative healthcare infrastructure” [(36), p.8]. In a more detailed manner, Creative Forces, a National Endowment for the Arts military healing arts network, developed a Strategic Framework and Five-Year Agenda which details three overarching objectives — develop multi-tiered leadership, develop organizational capacity, and generate research projects and processes — as well as benchmarks of success over a five-year timeline (33). Finally, the NIH Sound Health research plan explicitly details a set of research priorities which guide funding set out by its NIH and Kennedy Center partnership (34).

Most of the documents made a case at the intersection of arts and mental health and provided recommendations for progress. Of the recommendations which directly concerned a form of institutional or legislative policy, there were several thematic trends evident: (a) funding (*n* = 11); (b) federal and local efforts across sectors for sustained collective action (*n* = 9); (c) integrate the arts into current health initiatives (*n* = 7); (d) generalized arts and health advocacy (*n* = 6); (e) convene conversations (*n* = 4); (f) expand mental health infrastructure (*n* = 4); (g) other (*n* = 4); (h) expansion of current practices (*n* = 3); (i) national certifications (*n* = 2); and, (j) establish an arts and health continuum of services (*n* = 2). A detailed breakdown by article and category can be found in Appendix F.

Funding recommendations varied in scope and focus. Several centered funding more research on the benefits of arts for health (42, 57, 58), while other focused on funding for program development (45, 57, 58). Additionally, recommendations relevant to coverage, reimbursement, and incentivization were raised. For instance, the Rhode Island State Arts and Health Plan recommended studying existing national reimbursement models to examine insurer reimbursement opportunities (36). Additionally, ODPHP recommended maximizing “Medicaid and Medicare coverage to support evidence-based creative arts therapies for both mental and physical healthcare across a range of healthcare and community-based supportive art environments” [(35), p.108]. Incentivization was also raised as a viable avenue by National Assembly of State Arts Agencies (NASAA) (46) as they recommended that incentivization of arts and health cross-sector collaboration be established through additional credit points in federal grant programs such as community health programs funded by the US Department of Health and Human Services.

Efforts across sectors for sustained, collective action were discussed both locally and nationally through recommendations. Several documents aligned with this concept broadly; for instance, Sonke et al. (61) discussed establishing viable pathways which would enable cross-sectoral collaboration. Relative to recommendations with high specificity, organizations like the National Organization for Arts and Health recommended creating a national structure and a strategic plan for the intersection of arts and health to coalesce (59). Further, the American Art Therapy Association recommended reinstating the Presidents’ Committee on the Arts and Humanities and ensuring that there is newly instated mental health representation (42).

As it relates to integrating the arts into current health initiatives and convening conversations, there were several calls to action. Specific recommendations included state level plans which would integrate the arts into health policies (46), integrating the arts and health strategies into current efforts combatting the national the opioid epidemic (44), and even promoting the inclusion of creative arts therapies in national health military strategic agency as well as interagency initiatives (57). Further, relating to convening conversations, NOAH (55) recommended forming a speaker’s bureau which could then be mobilized to advance public awareness of the multitude of arts and health practices. Hanna et al. (32) recommended inviting the arts sector into both national and international conversations concerning the integration of wellbeing into policy development.

Recommendations also called on expanding upon current practices and establishing national certifications. More specifically, recommendations centered on expanding access to art therapy for veterans, active service members, and students within school settings (42). Expansion can also be considered as it relates to increasing the amount and types of policies which act to support creative arts therapy within both the Department of Defense and Veterans Administration (58). As it relates to credentials and certifications, NOAH (59) recommended establishing a nationally recognized credential for professional artists within healthcare settings. For more detail, a summary of recommended themes and their corresponding sources is available in Appendix E.

### Field evidence

All but three documents cited research literature. Those were a governmental executive order (20, 21) and a proclamation (37), as well as a working guide from Americans for the Arts which utilized case examples to narratively form their case (52). Further, of the included articles that were published after the landmark WHO Arts and Health review (6), about 46% (*n* = 6) utilized the seminal review as evidence (40, 46, 47, 49, 50, 54). Further, several documents highlighted a model, theory, or framework that guided the framing of the document. For instance, ODPHP (35) described how the Vital Conditions for Health and Well-Being Framework provided the document with an actionable, asset-based approach. National Assembly of State Arts Agencies (NASAA) (46) framed their case building for the arts around the U.S. surgeon general’s pillars of social connection and building community well-being (3). Additionally, several documents employed the Evidence-Based Framework for Using Arts in Public Health (48–50, 60). In addition to the evidence used, documents also articulated prevalent gaps in the literature. Notably, highlighted gaps included the lack of well-powered studies, longitudinal studies, and a public awareness of the intersection of arts and health.

### Funding and sustainability

As it pertains to funding and sustainability, the included documents discussed both concepts relative to the document’s discrete work as well as a trajectory for the field. Ten documents, equating to 34%, explicitly detailed how the document itself was financially supported (33, 36, 39, 46, 47, 51, 52, 54, 58, 61). Further, a trend regarding field funding was that of seeking multiple funding streams, both from the private and public sectors, to successfully scale work while also ensuring a sustainable funding model (32, 39, 41, 51, 53, 57, 58). Building on this concept, ODPHP (35) discusses the utility of braided funding, especially as it pertains to cross-sector collaboration. As it further relates to sustainability, included documents also peripherally contextualized impacts on other sectors. Notably, the most frequently mentioned was that of economic development (*n* = 13) (20, 21, 32, 33, 39, 41, 44, 45, 48–50, 52, 55, 57).

## Discussion

The compelling evidence for arts in health has catalyzed both national and international momentum for the field (6, 19–21). Concurrent to this momentum, calls from the field have advocated for the mobilization of policy as a sustainable strategy for the scaling of this work, including engagement of the arts to support mental health (19). As such, need has emerged to map and characterize existing arts in mental health policy. While a review of arts in public health policy internationally was conducted by Dow et al. (19), there is no current review focusing on the US or arts in mental health specifically. This scoping review sought to investigate what priorities and strategies are being employed in public health policies that seek to engage the arts to address mental health in the US. The evidence revealed several salient results: (a) the relative nascency of arts in mental health policy documents in the US; (b) that policy recommendations primarily center on creating sustained, collective action and leveraging funding; and (c) that the Arts sector alongside the Arts and Health sector are primarily leading policy work.

### Framing, strategies, and sustainability

This review identified both governmental or non-governmental policy documents, including legislative, regulative, litigative, or other documents. Across the included documents, about 24% were governmentally based. The documents included presidential and gubernatorial executive orders, as well as other policy documents that do not have the force of law, such as guidance documents produced by federal, state, or local agencies (18). These findings are in line with the view of arts and health policy in the US presented in the global review by Dow et al. (19).

The primary strategy employed by the reviewed documents was to make recommendations, which act as a precursory step toward legislative, regulative, or even litigative policies. As such, the current state of the arts in mental health policy in the US may be primed for advancement towards integrated policies such as those which have been mobilized in other countries. For instance, the Healthy Ireland Strategic Action Plan is a mental health plan that commits to strategic arts planning as an integral component of driving both public health and wellbeing in Ireland (19). As it relates to advancing these efforts in the United States, the WHO, in collaboration with the Jameel Arts and Health Lab, has launched the Healing Arts global outreach campaign which was featured alongside the United Nations General assembly in New York City (62).

The intentions described for mobilizing arts in mental health can be characterized by two primary trends in policy: creating sustained, collective action and leveraging funding. Regarding sustained, collective action, evident areas of momentum include creating viable pathways and infrastructure through which cross sector collaboration can occur. More specifically, recommendations also included creating a national structure and a strategic plan for the intersection of arts and health to coalesce as well as the reinstatement of the Presidents’ Committee on the Arts and Humanities accompanied with mental health representation (42, 59). These calls for action point to current voids in cross-sectoral practice which can be explained by rationale from the literature. For instance, Petchel et al. (63) describes that rather than an abundance of pathways or enablers for cross-sectoral collaboration, a lack of prioritized time for relationship building alongside risks to planned sustainability and compliance with funder or regulatory requirements can offset progress. Further, recommendations to integrate the arts into existing health initiatives resonate with international practices, such as social prescribing development in the UK (19, 64).

Pertaining to funding — a global concern as well (19) — some documents centered on funding research on the benefits of arts for health (42, 57, 58), while others focused on funding program development (45, 57, 58). Additionally, recommendations relevant to coverage, reimbursement, and incentivization were discrete, actionable recommendations set forth (35, 36, 46). These funding recommendations provide clear pathways by which cross-sectoral partnerships can begin to retain sustainability and collectively begin to address mental health nationally from a socioecological perspective. This overall directionality is also in line with the US-based arts and health caucus report which centers concepts such as integrating the arts into current health policies and increasing pathways for cross-sectoral collaboration at the municipal, state, and federal levels (65).

### Organizational domains

The arts as well as the arts and health sectors are the primary actors in advancing policy related to arts in mental health. Of the documents analyzed, the arts and arts and health sectors served as a primary author in the majority of the included documents while the health sector only served as a primary author in less than a third — a prevalent trend also evident globally (19). This trend can also be seen in the composition of national arts and health efforts such as the Interagency Working Group on Arts, Health, and Civic Infrastructure which has been convened by the arts sectors, the NEA, but also includes members such as HHS and the National Science Foundation, amongst others (12). In addition to the sectoral composition, it is also of note that across authorship, the documents were primarily of a national purview (*n* = 26) while only a few considered a local or state frame (36, 37, 39). This exemplifies the high prevalence of “top down” strategies for arts in mental health policy in the US (66). However, it is of note that the literature contends that while a “top down” policy approach offers the benefit of guiding the system, “bottom up” approaches are also essential as they provide opportunities to amplify local voices (66). Another important consideration in this amplification is that of equitable practice. About 55% of included documents either directly mentioned equity as a central component of the document or had a primary focus on equitable practice as evidenced by language throughout the document. By providing an equitable lens to health policies, the field can collectively ensure that minoritized populations are supported and that these policies do not inadvertently create disparities (67).

### Strengths, limitations, and recommendations

There were evident strengths and limitations to this review. A primary strength was the alignment between the approach of employing a scoping review with the exploratory nature of the research question (68). As such, the broad scope of evidence that scoping reviews permit allowed for the inquiry to fully capture relevant data. Additionally, this review engaged at least two reviewers per document at each selection stage, which increased the number of relevant documents identified during the screening process thus limiting both information and selection bias (69). Further, an in-depth grey literature search was conducted across 16 locations inclusive of repositories, databases, and search engines which acted to increase the rigor of the search (70). In addition to this hand search, in accordance with the international arts and health policy review undertaken by Dow et al. (19), a survey was disseminated to further capture any evidence which may have been missed in the traditional search. This strategy further bolstered the comprehensiveness of the search. Several limitations were also present. Notably, there was no quality or risk of bias assessment which would have further ensured the credibility of the search results. Additionally, due to the nascency of arts in mental health policy in the US, the study considered documents with a range of primary foci as few were squarely centered on arts and mental health discretely. None of the included documents were clear regarding the modes of arts participation, which limits understanding of how different forms of engagement may relate to mental health outcomes. Lastly, many of the recommendations included may be pragmatic rather than evidence-based, which could limit their potential impact. As arts in mental health policy in the US advances, several considerations should remain at the forefront. First, this review has identified key areas of momentum as it pertains to recommendations, organizations engaged, and priority mental health topics. With that, future work should intentionally build on areas of momentum to effectively catalyze future efforts. For instance, the alignment of these findings with the US Surgeon General’s advisory on the epidemic of loneliness and social isolation may present an opportunity for synergistic work (3). This further offers an opportunity for empirical evidence to be incorporated earlier in the policy development cycle, further supporting evidence-based decision making. Second, stakeholders should consider efforts to progress policy types at this intersection by seeking to develop legislative, regulative, or even litigative cross-sectoral, arts in mental health policies. Third, current momentum in the US offers a “policy window” as there is alignment, as evidenced in this review, amongst national policy makers, the prevailing mental health crisis, and opportunities for arts in mental health policies as a viable solution (22). However, it is important to consider that the current administration has made actionable steps toward undermining arts infrastructure which had seen bipartisan support across past administrations — weakening a once very open policy window (23, 24). More specifically, the Trump administration has challenged arts infrastructure by both disbanding the US President’s Committee on Arts & Humanities as well as removing the Kennedy Center’s social and impact team as well as replacing President Deborah Rutter after her decade of service with himself (23, 24). Subsequently, while there are top-down approaches underway to seize this window, the current political context reinforces the need for bottom-up policy approaches which are directly informed at a local level (66). Subsequently, it becomes essential to understand how to best engage or promote the engagement of local artists, mental health practitioners, arts in mental health researchers, and policy makers in the development of arts in mental health policies moving forward.

## Conclusion

As the US seeks to effectively address the complexity of the mental health crisis, it is imperative to engage cross-sectoral strategies, including those of arts in mental health. However, to sustainably scale arts in mental health efforts, infrastructure and policy are necessary. As evidenced by the current arts in mental health policy literature, policy recommendations from the field primarily center on creating sustained, collective action and leveraging funding. However, the arts sector alongside the arts and health sector are mainly leading policy work, and the current arts in mental health policy documents are relatively nascent. To effectively scale policy at this intersection there needs to be a conscious effort to further engage the health sector; promote the development of legislative, regulative, or even litigative arts in mental health policies; and catalyze bottom-up policy approaches which arise from a local level. It also remains essential to consider that policy at this intersection provides a lever to support health equity and ethical imperatives which are of particular importance in light of ongoing disparities in mental health outcomes. As we consider paths forward, this is the time to collaborate and co-vision as “policy is an imagining of the future” [(39), p.1].
