# Supplementary material for: A scoping review of arts in mental health policy in the United States
Source: Front Public Health. 2025 May 5;13:1562990. doi: 10.3389/fpubh.2025.1562990 (PMC12097218; doi:10.3389/fpubh.2025.1562990)
Supplement: Supplementary file 1 [file Data_Sheet_1.pdf]

Appendix A

PubMed, 3/22/2024. With English language limit. 760 results
Results: 760. 760 files uploaded to Covidence
United States

Title/Abstract(“united states” OR “USA” OR appalachia* OR “great lakes” OR midatlantic OR “mid-atlantic” OR “mid atlantic” OR midwest* OR “mid west” OR “mid-west” OR “mid western” OR “mid-western” OR “mid-westerner” OR “mid-westerners” OR “mid westerner” OR “mid westerners” OR Alaska* OR inuit* OR inupiat* OR inupiak* OR inupiaq* OR aleu* OR “na dene" OR “na-dene" OR eskimo* OR hawaii* OR washington* OR oregon* OR california* OR idaho* OR nevada* OR nevadian* OR utah* OR arizona* OR arizonian* OR montana* OR wyoming* OR colorad* OR “new mexico” or “new mexican” OR “new mexicans” OR dakota* OR nebraska* OR kansas* OR kansan* OR oklahoma* OR texas* OR texan* OR minnesota* OR iowa* OR iowegian* OR missouri* OR arkansa* OR louisiana* OR cajun* OR wiscons* OR illin* OR kentuck* OR tennesse* OR mississippi* OR alabam* OR florida* OR floridian* OR michigan* OR indiana* OR ohio* OR virginia* OR carolina* OR carolinia* OR georgia* OR maine* OR “new hampshirite” OR “new hampshirites” OR vermont* OR massachusetts* OR “new york” OR “new yorker” OR “new yorkers” OR “rhode island” OR “rhode islander” OR “rhode islanders” OR connecticut* OR “new jersey” OR “new jerseyan” OR “new jerseyans” OR jerseyite* OR delaware* OR pennsylvania* OR maryland* OR “district of columbia” OR "pacific northwest" OR "pacific northwestern" OR "pacific northwesterner" OR "pacific northwesterners" OR “American Samoa*” OR “Guam” OR “Northern Mariana Islands” OR “Puerto Rico” OR "U.S. Virgin Islands” OR “US Virgin Islands” OR “United States Virgin Islands”) OR ("United States"[Mesh])

Arts (SEARCH IN TITLE ONLY)

Title: arts OR artist OR artists OR artistic OR artistry OR quilt* OR printmak* OR silkscreen* OR "silk screen*" OR acting OR actor OR actors OR actress OR actresses OR playwrit* OR jazz OR "art-based" OR "arts-based" OR creativity OR "creative effort" OR “creative efforts” OR "creative engagement" OR "creative expression" OR “creative expressions” OR "creative process" OR “creative processes” OR "creative practice" OR “creative practices” OR "creative therapy" OR "creative therapies" OR "creative writing" OR "group writing" OR "entertainment education" OR "expressive activity" OR “expressive activities” OR "expressive writing" OR "graphic novel" OR "graphic novels" OR "journal writing" OR "diary writing" OR "movement expression" OR "movement therapy" OR “movement therapies” OR roleplay* OR animation OR animations OR artwork OR artworks OR ballet OR caricature OR caricatures OR cartoon OR cartoons OR choir OR choirs OR chorister OR choristers OR choral OR choreograph* OR clay OR collage OR collages OR collaging OR comic OR comics OR comicbook OR danc* OR drama OR dramatic OR psychodrama OR drawing OR drawings OR edutainment OR film OR films OR filming OR fotonovela OR fotonovelas OR photonovella OR photonovellas OR "folk media" OR improvisation OR improvisations OR improvization OR improvizations OR journaling OR mandala OR mandalas OR mural OR murals OR museum OR museums OR music OR musical OR musician OR musicians OR novela OR novelas OR novella OR novellas

OR opera OR operas OR operatic OR paint* OR photograph* OR photovoice OR "photo voice" OR pictorial OR plays OR poet OR poets OR poetry OR poem OR poems OR haiku* OR portrait OR portraits OR portraiture OR potter OR potters OR pottery OR puppet OR puppets OR puppetry OR puppeteer OR puppeteers OR puppeteering OR rap OR raps OR rapping OR sculpt OR sculpts OR sculpture OR sculptures OR sculpting OR sculpted OR sing OR singing OR sings OR singer OR singers OR skit OR skits OR song OR songs OR “story circle” OR stories OR storyline OR storylines OR storytell OR storyteller OR storytellers OR storytelling OR textile OR textiles OR theatr* OR theater* OR transmedia OR watercolor* OR watercolour* OR "water-color*" OR "water-colour*" OR ceramics OR mosaic OR mosaics OR lyrics OR graffiti OR "hip hop" OR "hip-hop" OR aesthetic* OR esthetic* OR "spoken word" OR sketch* OR coloring OR colouring OR video* OR movie* OR webcast* OR “motion pictures” OR cinema* OR gallery OR galleries OR "virtual reality" OR “virtual realities” OR "narrative therapy" OR “narrative therapies” OR “digital culture” OR sew OR sews OR sewing OR weave OR weaves OR weaving OR crochet OR crochets OR crocheting OR knit OR knits OR knitting OR spinning OR needlework OR needlepoint* OR macram* OR embroider* OR "rug hooking" OR tapestry OR tapestries OR dyeing OR "tie-dy*" OR temari OR shibori OR paperfold* OR "paper folding" OR origami OR scrapbook* OR stamping OR decoupage OR calligraphy OR papercutting OR "paper cutting" OR papercraft* OR paperart* OR craft OR crafts OR crafting OR quilling OR papermaking OR "paper making" OR printmaking OR marbling OR screenprint* OR "screen printing" OR "paper mache" OR "papier mache" OR illustration OR illustrations OR whittling OR woodcarving OR carving OR "wood work*" OR woodwork OR woodworks OR woodworking OR woodturning OR coopering OR cooperage OR woodburn OR woodburning OR pyrography OR inlay OR enameling OR cloisonn* OR engraving OR engravings OR embossing OR embossings OR etching OR etchings OR "wire work*" OR "metal work*" OR metalwork* OR blacksmith* OR smithing OR tinsmith* OR goldsmith* OR silversmith* OR beading OR handbuilding OR "hand building" OR "glass blowing" OR glassblowing OR "stained glass" OR basketmak* OR basketry OR ikebana OR "flower arrang*" OR "floral arrang*" OR "leather work*" OR leatherwork* OR batik* OR lithograph* OR "jewelry mak*" OR jewelrymak* OR stitchery OR handicraft* OR sandplay* OR "sand play*" OR sandpaint* OR decorat* OR "art-therapy” OR “art-therapies” OR “arts-therapy” OR “arts-therapies” OR “drama-therapy” OR “dance-therapy” OR “dance-therapies” OR “music-therapy" OR “music-therapies” OR gardening OR beadwork OR featherwork OR “feather-work” OR flameworking

OR "Art"[Mesh:NoExp] OR "Caricatures as Topic"[Mesh] OR "Engraving and Engravings"[Mesh] OR "Motion Pictures"[Mesh] OR "Paintings"[Mesh] OR "Pictorial Works as Topic"[Mesh] OR "Portraits as Topic"[Mesh] OR "Sculpture"[Mesh] OR "Music"[Mesh] OR "Creativity"[Mesh] OR "Drama"[Mesh] OR "Pictorial Work" [Publication Type] OR "Psychodrama"[Mesh] OR "Dancing"[Mesh] OR "Museums"[Mesh] OR "Photography"[Mesh:NoExp] OR "Poetry as Topic"[Mesh] OR "Portrait" [Publication Type] OR "Comic Books as Topic"[Mesh] OR "Narration"[Mesh] OR "Esthetics"[Mesh] OR "Video-Audio Media" [Publication Type:NoExp] OR "Narrative Therapy"[Mesh] OR "Sensory Art Therapies"[Mesh] OR "Gardening"[Mesh] OR "Books, Illustrated"[Mesh] OR "Diaries as Topic"[Mesh] OR "Textiles"[Mesh] OR "Ceramics"[Mesh] OR "Personal Narratives as Topic"[Mesh] OR "Personal Narrative" [Publication Type]))

NOT (antiretroviral OR “anti retroviral” OR “anti-retroviral” OR “HAART” OR “assisted reproductive therapy” OR “assisted reproductive therapies” OR “state of the art” OR "Antiretroviral Therapy, Highly Active"[Mesh] OR "Reproductive Techniques, Assisted"[Mesh])

Policy

Title/Abstract: "Policy" OR "policies" OR "legislat*" OR "regulat*" OR "national fund*" OR "nationally fund*" OR "funding" OR "funded" OR "law" OR "laws" OR "rule" OR "rules" OR "ruling" OR "ruled" OR "state policy" OR "state policies" OR "State legislat*" OR "state regulation*" OR "state-funded" OR "state fund" OR "state funding" OR "state funds" OR "state law" OR "state laws" OR "state rule" OR "state ruled" OR "state rules" OR "amendment*"OR "statute*" OR "state interest*" OR “national plan” OR “national plans” OR “national program*” OR “national strateg*” OR “state regulat*” OR “statutory law” OR “statutory laws” OR “litigation” OR “report” OR “reports”

OR ("Policy"[MeSH Terms] OR "Legislation as Topic"[MeSH Terms] OR "Government Regulation"[MeSH Terms] OR "Jurisprudence"[MeSH Terms]

Mental Health

Title/Abstract: ("mental health" OR depress* OR depressiv* OR anxiety OR anxious* OR suicid* OR "self harm*" OR "self-harm*" OR "alcohol drinking" OR "substance us*" OR "substance abuse" OR insecur* OR anger OR angry OR angriness OR isolat* OR antisocial* OR "social withdrawal" OR "attention deficit disorder" OR "attention deficit disorders" OR "attention deficit hyperactivity disorder" OR "attention deficit hyperactivity disorders" OR "eating disorder*" OR "body dysmorphi*" OR bipolar OR schizophreni* OR bulimi* OR anorexi* OR "mental condition" OR “mental conditions” OR "mental distress" OR “mental ill health” OR “mentally unwell” OR “mood disorder” OR “mood disorders” OR “psychic health” OR “affective disorder” OR “affective disorders” OR “dementia praecox” OR “self injur*” OR “self-injur*”OR “neurocirculatory asthenia*” OR “panic disorder” OR “panic disorders” OR “panic attack” OR “panic attacks” OR “obsessive-compulsive disorder” OR “obsessive-compulsive disorders” OR “obsessive compulsive disorder” OR “obsessive compulsive disorders” OR “OCD” OR “obsessive compulsive neurosis” OR “obsessive-compulsive neurosis” OR “mental disorder” OR “mental disorders” OR “psychiatric disorder” OR “psychiatric disorders” OR “psychiatric disease” OR “psychiatric diseases” OR “mental illness” OR “mental illnesses” OR “mentally ill” OR “psychotic disorders” OR “PTSD”)

English language filter.

Appendix B

Data Extraction Instrument

Data Type	Data
Mental Health topic	
Art Mode(s)	
Art Form(s)	
Leading Formal or Informal Partnership(s)	
Funding Mechanisms	
Equity Lens	
Theoretical Approach	
Primary evidence that informed policy	
Plan for sustainability	
Intended Outcomes	
Potential impacts of the policy on other sectors and high priority issues (e.g., sustainability, economic impact)	
Intended audience	
Associated Costs	
Proposed Timeline(s) for implementation	
Any gaps in evidence base	
Priorities	
Strategies employed	
Organization domain	
Primary and Secondary Foci	
Document Type	

Appendix C

**Organization**	**References**
Americans for the Arts	Americans for the Arts, 2018; Atkins & Jacobson Blumenfeld, 2020; Bivens, 2013; Rollins, 2012
ArtPlace America	for Arts in Medicine, 2020a; University of Florida Center for Arts in Medicine, 2020b; University of Florida Center for Arts in Medicine, 2020c
John F. Kennedy Center for the Performing Arts	Cheever et al., 2018; Edwards et al., 2023; National Institute of Health, 2018
National Assembly of State Arts Agencies	National Assembly of State Arts Agencies, 2017; National Assembly of State Arts Agencies, 2019; National Assembly of State Arts Agencies, 2024; University of Florida Center for Arts in Medicine, 2020b
National Center for Creative Aging	Boyer, 2007; Hanna, 2011; Hanna et al., 2015
National Endowment for the Arts	Hanna, 2011; Hanna et al., 2015; Iyengar et al., 2018; Office of Disease Prevention and Health Promotion, 2022
National Institute of Health	Cheever et al., 2018; Edwards et al., 2023; National Institute of Health, 2018
National Organization of Arts and Health	National Organization of Arts and Health, 2017; National Organization of Arts and Health, 2018; National Organization of Arts and Health, 2020
U.S. Department of Health & Human Services	Hanna, 2011; Office of Disease Prevention and Health Promotion, 2022
University of Florida Center for Arts in Medicine	Pesata et al., 2020; Sonke et al., 2019; University of Florida Center for Arts in Medicine, 2020a<br>University of Florida Center for Arts in Medicine, 2020b<br>University of Florida Center for Arts in Medicine, 2020c

Appendix D

Across the briefs and reports category, the Arts sector was engaged as a primary organizational author across all the briefs (n=9) while the second most represented organizational domain as a primary author was that of Arts and Health (n=6) followed by Health (n=4). Of the documents which were classified as reports (n=9), generalized reports, white papers, and a blueprint for action were represented (Atkins & Jacobson Blumenfeld, 2020; Cheever et al., 2018; Edmonds et al., 2017; Hanna et al., 2011; National Organization of Arts and Health [NOAH], 2017; NOAH, 2020; NOAH, 2018; Rollins, 2012; Sonke et al., 2019). As it pertained to organizational domains represented as primary contributors to the document, Arts and Health was most represented (n=7) while the Arts sector was the second most represented (n=5). Regarding toolkits, guides, plans, and agendas, the Arts sector was represented across all three, whereas Arts and Health, as well as the Health sector, were each only represented once. Of the documents which were considered as either plans or agendas (n=4), there was a state plan (RIDOH & RISCA, 2019), a federal plan (ODPHP, 2022), a strategic framework and agenda (Iyengar et al., 2018), as well as a research plan (NIH, 2018). Additionally, the Arts sector was represented as a primary author category across all plans or agendas (n=4) while Health was represented across half (n=2). In reference to the documents which did not adhere to a category, the Arts sector was represented across three of these documents (Hanna et al., 2015; Harlow, 2021; Solis, 2024) while Municipal Leadership was represented across two (Biden, 2022; Solis, 2024). Finally, only one had the Arts and Health sector represented (Hanna et al., 2015).

Appendix E

Article	Arts	Public Health	Funders/Investors	Healthcare/Clinical Research	Policy Makers/Government	General Public	Education	Military	Other
American Art Therapy Association, 2021					X				
Americans for the Arts, 2018					X	X			
Atkins & Jacobson Blumenfeld, 2020	X		X	X	X			X	
Biden, 2022						X			
Bivens, 2013						X		X	
Boyer, 2007	X		X	X					X
Cheever et al., 2018	X			X					
Edmonds et al., 2017	X	X	X		X				X
Edwards et al., 2023	X			X					X
Hanna et al., 2011					X	X			X
Hanna et al., 2015					X				X
Harlow, 2021				X			X		
Iyengar et al., 2018				X					
National Assembly of State Arts Agencies, 2017	X		X		X		X		X
National Assembly of State Arts Agencies, 2019	X								
National Assembly of State Arts Agencies, 2024	X	X		X	X	X			
National Institute of Health, 2018	X			X			X		
National Organization of Arts and Health, 2017	X			X	X		X		
National Organization of Arts and Health, 2018	X			X	X		X		X
National Organization of Arts and Health, 2020	X			X	X				X
Office of Disease Prevention and Health Promotion, 2022					X				X
Pesata et al., 2020	X								
Rhode Island Department of Health & The Rhode Island State Council on the Arts, 2019	X	X		X	X	X			X
Rollins, 2012	X								
Solis, 2024						X			
Sonke et al., 2019	X	X	X		X		X		X
University of Florida Center for Arts in Medicine, 2020a		X							
University of Florida Center for Arts in Medicine, 2020b		X		X	X		X		X
University of Florida Center for Arts in Medicine, 2020c		X							

Appendix F

Policy Recommendations by Theme

Generalized Arts and Health Advocacy (n=6)

- Catalyze community, move policy, shift culture (Edmonds et al., 2017, p. 71).
- Call to action to focus on solutions for arts and health (Americans for the Arts, 2018).
- Increasing advocacy (National Organization of Arts and Health, 2020).
- “Advancing cultural diplomacy” (Bivens, 2013, p. 7-9).
- "Develop an advocacy platform in partnership with other advocacy groups present such as Americans for the Arts to influence policymakers across the health and wellness spectrum services” (National Organization of Arts and Health, 2018, p. 12).
- “Speak in one voice” (Rollins, 2012, p. 8).

Federal and Local Efforts Across Sectors for Sustained Collective Action (n=9)

- “Leverage[s] the Vital Conditions for Health and Well-Being Framework (the Vital Conditions Framework) to guide and orchestrate federal efforts across sectors and focus areas for greater sustained collective action” (Office of Disease Prevention and Health Promotion, 2022, p. 14).
- “Shift[s] the way federal departments and agencies build partnership and collaboration with state, local, and cross-sector community-based organizations to amplify collective resources and strengthen systems that enable well-being” (Office of Disease Prevention and Health Promotion, 2022, p. 14).
- “Establish a federal interagency task force” (Hanna et al., 2011, pp. 9, 30).
- "Provide immediate pathways for enabling cross-sector collaboration" (Sonke et al., 2019, p. 41).
- "Reinstate the Presidents' Committee on the Arts and Humanities and ensure mental health representation" (American Art Therapy Association, 2021, p. 3).
- “Creation of a new national structure and strategy for the arts, health, and well-being arena to coalesce” (National Organization of Arts and Health, 2017, p. 41).
- “Promote increased inter-agency and private sector support” (Rollins, 2012, p. 7).
- “Develop art and cultural policies that nurture accessibility and inclusive access, including the design of built environments and services that support programmatic inclusion (e.g., sign language and audio description), leveraging cross-agency collaboration to identify opportunities to integrate such policies in all sectors" (Office of Disease Prevention and Health Promotion, 2022, p. 62).
- “Expand existing coordination between HUD and NEA to institutionalize the incorporation of design and art into all new developments, with a priority to engage design and art from within the local community" (Office of Disease Prevention and Health Promotion, 2022, p. 147).

Funding (n=11)

- “Identifies opportunities to maximize federal steady-state resources within current agency authority to strengthen resilience” (Office of Disease Prevention and Health Promotion, 2022, p. 14).

- "Fund more research on the benefits of art therapy across populations and diagnoses" (American Art Therapy Association, 2021, p. 3).
- Increase funding from state, federal and private funders for the arts in rural and urban communities across the nation (National Assembly of State Arts Agencies, 2017).
- "Promote increased interagency and private sector support and expedite funding for research" (Atkins & Jacobson Blumenfeld, 2020, p. 27).
- "Increase policies that provide for the support of creative arts therapists within the DoD and VHA" (Atkins & Jacobson Blumenfeld, 2020, p. 27).
- "Encourage increased public and private sector funding for program development, implementation, and evaluation, and bringing successful programs to scale" (Atkins & Jacobson Blumenfeld, 2020, p. 27).
- “Expedite funding for research” (Rollins, 2012, p. 7).
- “Encourage increased public and private sector funding for program development, implementation, and evaluation, and bringing successful programs to scale” (Rollins, 2012, p. 8).

Coverage/Reimbursement/Incentivization

- “Conduct a study of existing national reimbursement models and examine the potential for insurer reimbursement in Rhode Island" (Rhode Island Department of Health & The Rhode Island State Council on the Arts, 2019, p. 11).
- “Maximize Medicaid and Medicare coverage to support evidence-based creative arts therapies for both mental and physical healthcare across a range of healthcare and community-based supportive art environments" (Office of Disease Prevention and Health Promotion, 2022, p. 108)
- “Incentivize arts and health cross-sector collaboration through extra-credit points in federal HHS grant programs, like grants for community health programs.” (National Assembly of State Arts Agencies, 2024, p. 6).

Convene Conversations (n=4)

- “Convene a series of technical workshops to help develop research proposals” (Hanna et al., 2011, p. 10).
- “Convene a policy-setting body to coordinate and implement the priorities identified in this Plan” (Rhode Island Department of Health & The Rhode Island State Council on the Arts, 2019, p. 11).
- “Form a speaker’s bureau, used to enhance communication and public awareness between all the sectors in health care and related services (National Organization of Arts and Health, 2018, p. 12).
- “Bring the arts to national and international conversations about integrating the concept of well-being into policy development” (Hanna et al., 2011, pp. 10, 31).

Expansion of Current Practices (n=3)

- "Expand access to art therapy for our veterans, service members, and their families" (American Art Therapy Association, 2021, p. 2).
- "Expand the workforce of art therapists in schools" (American Art Therapy Association, 2021, p. 2).

- “Increase policies that provide for the support of creative arts therapists within the Department of Defense and Veterans Administration" (Rollins, 2012, p. 7).

National Certifications (n=2)

- “Nationally established credentialing or certification is needed for professional artists to work in healthcare settings, for whom no standard of additional training beyond artistic competency currently exists” (National Organization of Arts and Health, 2017, p. 22)
- Formation of National Standards, Training, and Certification of Professional Artists, Healthcare Arts Administrators, and Healthcare Arts Consultants" (National Organization of Arts and Health, 2017, p. 42)

Integrate the Arts into Current Health Initiatives (n=7)

- “Develop state level plans to integrate arts and creativity into health policy and programs" (National Assembly of State Arts Agencies, 2024, p. 6).
- Integrate the arts into efforts to combat the opioid epidemic by supporting addiction recovery and prevention through arts-based therapies and creative engagements (National Assembly of State Arts Agencies, 2019, p. 5).
- “Advocate for inclusion of arts and culture in the HHS's Healthy People 2030" (National Assembly of State Arts Agencies, 2024, p. 6)
- “Promote the inclusion of the arts and creative arts therapies in national health and military strategic agency and department plans and inter-agency initiatives” (Rollins, 2012, p. 7).
- “Use research findings to align arts and health policy and practice with RIDOH’s 23 statewide population health goals and other key state policies" (Rhode Island Department of Health & The Rhode Island State Council on the Arts, 2019, p. 11).
- "Promote the inclusion of the arts and creative arts therapies in national health and military strategic agency and department plans and interagency initiatives" (Atkins & Jacobson Blumenfeld, 2020, p. 27).
- **“**Incentivize strengthening and broadening community care beyond basic services to include additional supports for well-being and social connection, including arts and creativity" (National Assembly of State Arts Agencies, 2024, p. 6).

Expand Mental Health Infrastructure (n=4)

- "Expand and develop the mental health workforce" (American Art Therapy Association, 2021, p. 1).
- "Permanently expand access to and ensure parity for teletherapy” (American Art Therapy Association, 2021, p. 1).
- "Offer mental health services to detained individuals and reunite separated children with their families" (American Art Therapy Association, 2021, p. 2).
- "Shift resources and response from law enforcement to mental health practitioners during mental health crises" (American Art Therapy Association, 2021, p. 2).

Establish an Arts and Health Continuum of Services (n=2)

- “Develop and promote an arts and health continuum of services, including the use of creative arts therapies, therapeutic arts, and arts for educational and expressive purposes"

(Rhode Island Department of Health & The Rhode Island State Council on the Arts, 2019, p. 11).
- “Delineate an 'Arts & Health in the Military' continuum of services including the use of creative arts therapies, therapeutic arts, and arts for educational and expressive purposes” (Rollins, 2012, p. 8).

Other (n=4)
- “Recognize that artists rendering these services are valued professionals” (Rollins, 2012, p. 8).
- “Orients federal support to prioritize understanding community-driven needs and prioritize tailored solutions” (Office of Disease Prevention and Health Promotion, 2022, p. 14).
- “Support bringing together local arts communities with service members, veterans, and their families” (Rollins, 2012, p. 8).
- Work toward a broad public relations campaign using arts in health to affect change in the public’s perception of community health services to encourage engagement and draw people in from the shadows. (National Organization of Arts and Health, 2018)
